# Heterogeneous Disease Progression in a Mouse Model of Vascular Cognitive Impairment

**DOI:** 10.3390/ijms21082820

**Published:** 2020-04-17

**Authors:** Na Kyung Lee, Hunnyun Kim, Jehoon Yang, Jeyun Kim, Jeong Pyo Son, Hyemin Jang, Duk L. Na

**Affiliations:** 1 School of Medicine, Sungkyunkwan University, 81 Irwon-ro, Gangnam-gu, Seoul 06351, Korea; nakyunglee@skku.edu; 2 Stem Cell & Regenerative Medicine Institute, Samsung Medical Center, 81 Irwon-ro, Gangnam-gu, Seoul 06351, Korea; hmjang57@gmail.com; 3 Samsung Alzheimer Research Center, Samsung Medical Center, 81 Irwon-ro, Gangnam-gu, Seoul 06351, Korea; 4 Laboratory Animal Research Center, Samsung Biomedical Research Institute, 81 Irwon-ro, Gangnam-gu, Seoul 06351, Korea; mdpkhn@gmail.com (H.K.); jehoon815.yang@samsung.com (J.Y.); jeyun604.kim@sbri.co.kr (J.K.); 5 Laboratory Animal Center, Osong Medical Innovation Foundation, Cheongju 28160, Korea; jpyo.son@gmail.com; 6 Department of Neurology, Samsung Medical Center, Sungkyunkwan University School of Medicine, 81 Irwon-ro, Gangnam-gu, Seoul 06351, Korea; 7 Neuroscience Center, Samsung Medical Center, 81 Irwon-ro, Gangnam-gu, Seoul 06351, Korea; 8 Department of Health Sciences and Technology, SAIHST, Sungkyunkwan University, 81 Irwon-ro, Gangnam-gu, Seoul 06351, Korea

**Keywords:** vascular cognitive impairment, reproducibility, ameroid constrictor, microcoil, heterogeneity

## Abstract

Recently, an asymmetric vascular compromise approach that replicates many aspects of human vascular cognitive impairment (VCI) has been reported. The present study aimed to first investigate on the reproducibility in the disease progression of this newly reported VCI model using wild-type C57BL6/J mice. The second aim was to assess how this approach will affect the disease progression of transgenic Alzheimer’s disease (AD) 5XFAD mice subjected to VCI. C57BL6/J and 5XFAD mice were subjected to VCI by placing an ameroid constrictor on the right CCA and a microcoil on the left CCA. Infarcts and hippocampal neuronal loss did not appear predominantly in the right (ameroid side) as expected but randomly in both hemispheres. The mortality rate of C57BL6/J mice was unexpectedly high. Inducing VCI reduced amyloid burden in the hippocampi of 5XFAD mice. Since VCI is known to be complex and complicated, the heterogeneous disease progression observed from this current study shares close resemblance to the clinical manifestation of VCI. This heterogeneity, however, makes it challenging to test novel treatment options using this model. Further study is warranted to tackle the heterogeneous nature of VCI.

## 1. Introduction

Cognitive impairment or dementia caused by cerebrovascular injury is referred to as vascular cognitive impairment (VCI). Second to Alzheimer’s disease (AD), VCI is the most common cause of cognitive impairment in the elderly population. VCI is heterogeneous depending on the pathophysiology and distribution of cerebrovascular injury [1,2]. The pathogenesis of VCI is multifactorial. Common pathology underlying VCI include thromboembolism and hypoperfusion. Complexity worsens when vascular changes occur concurrently with AD pathology thus creating a mixed pathology. It is known that a combination of both AD and cerebrovascular pathology is relatively more common while pure AD or pure vascular dementia is not as prevalent [3,4].

Various models have been used to study VCI in rodents [5]. Unlike global ischemia models that severely disrupt blood flow via the two-vessel occlusion (2VO) technique, VCI due to hypoperfusion is generated by chronically reducing the blood flow of the common carotid artery (CCA) [6]. These animal models, however, are unable to fully recapitulate the complex features of human VCI. Recently, a group has introduced a mouse model that closely recapitulates major aspects of human subcortical VCI [7]. This model involves the asymmetric application of a microcoil to the left CCA and an ameroid constrictor to the opposite, right CCA. The ameroid constrictor comprises a hygroscopic casein within the titanium jacket as an internal lumen. The casein expands when exposed to water and thus arterial stenosis occurs as time progresses. The extent of brain tissue damage appears to differ depending on the diameter of the ameroid constrictor [8]. Along with a gradual reduction in cerebral blood blow (CBF) and cerebral infarcts in the subcortical regions, impairment in both motor function and working memory were also manifested from this model.

In comparison to a relatively greater number of studies that have used the bilateral CCA stenosis (BCAS)/occlusion method [9,10,11], many studies have not utilized this asymmetric vascular compromise approach in their studies. A drawback of one BCAS model, which involves the bilateral application of microcoils, is the acute CBF drop followed by recovery and absence of cerebral infarcts, which does not faithfully represent the features of human VCI [7,12]. Other than using microcoils, many groups have also used silk/nylon threads or clamps to ligate the arteries or even the CCA stump [13,14]. Based on the approach taken, the manifestation of the disease seems to differ among the models, and most importantly only partially recapitulates the complicated nature of human VCI.

Considering that multiple mouse models are generally recommended to investigate clinically heterogeneous diseases [15], based on the features exhibited from the recently reported asymmetric vascular compromise approach, this model is deemed to have high clinically translational potential. The reproducibility of this approach, however, has not been investigated more closely. The objectives of this present study were to first assess reproducibility in the disease progression of mice subjected to asymmetric vascular compromise (left CCA-microcoil, right CCA-ameroid constrictor). As reported previously, we expected that histopathological manifestations such as cortical and subcortical infarcts as well as hippocampal neuronal loss would be observed on the ameroid constrictor (right side) of the mouse brain, whereas white matter lesions would be detected on the microcoil side (left side). Furthermore, reports have been made vascular pathology coexists with AD pathology [16,17] and that vascular dysregulation and pathology can accelerate the pathogenesis of AD [18]. Based on currently available animals, it is difficult to simultaneously investigate both on AD and chronic hypoperfusion features from a single transgenic mouse model. Thus, our second objective was to evaluate whether it would be feasible to observe changes in AD pathogenesis by subjecting a transgenic AD mouse model to asymmetric vascular compromise.

## 2. Results

### 2.1. The Mortality Rate of C57VCI Mice Was High

Out of the 17 mice that underwent VCI surgery (Figure S1), only six survived up to the post 32-day endpoint (C57VCI 1, 2, 5, 6, 11, and 12). All the sham operated mice (*n* = 3) survived up to 32 days. Based on the Kaplan Meier survival analysis, the survival rate of the C57VCI group was 27.3% (Figure S1). There were four mice (out of 17) that died within a week after performing VCI surgery. A majority of the C57VCI mice (eight out of 17) died within 8 to 29 days. 

### 2.2. C57VCI Mice Displayed a Wide Variation in Development of Infarcts

**Figure 1:**
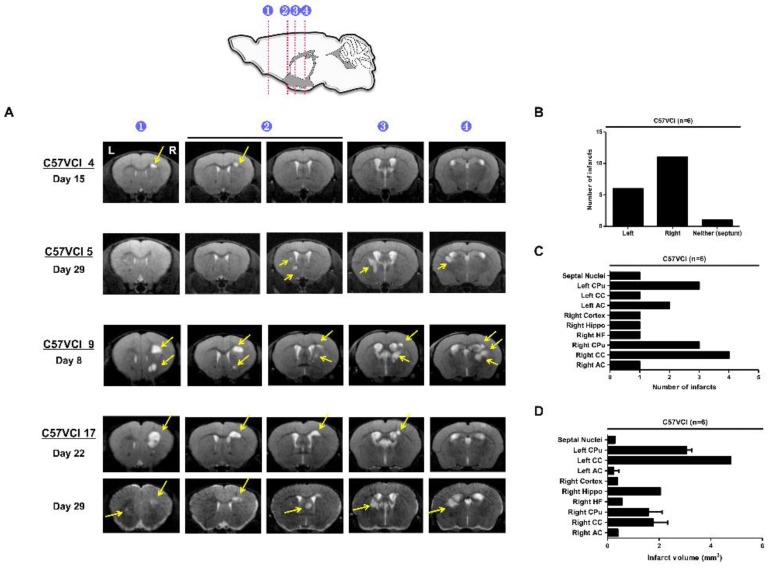
Variation in appearance and volume of cerebral infarcts of the C57VCI mice. (**A**) Representative T2 weighted MR images of 4 C57 mice subjected to VCI (top to bottom: C57VCI 4, 5, 9, 17). Cerebral infarcts appeared at 4 different time points (Day 15, 22, 8, and 29, respectively) for each of the mice. Hyperintense signals (solid yellow arrows) indicate the location of infarcts. L indicates left and R indicates the right hemisphere of the mouse brain. The numbers on the sagittal section of the mouse brain (top illustration) indicate the localization of cerebral infarcts in representative areas of the C57VCI parenchyma: (1) forceps minor (2) external capsule of the corpus callosum (3) caudate putamen, and (4) hippocampal fimbria. (**B**) Total number of infarcts observed from 6 of the 17 C57VCI mice (C57VCI 4, 5, 9, 15, 16, 17). (**C**) Distribution of infarcts. CC = corpus callosum, CPu = caudate putamen, AC = anterior commissure, Hippo = hippocampus, and HF = hippocampal fimbria. (**D**) Volume of the cerebral infarcts (mm^3^).

Magnetic resonance imaging (MRI) was utilized to assess infarct characteristics such as progression over time and volume. T2 weighted MR images were acquired weekly (post 8, 15, 22, and 29 days) following VCI surgery (Figure S1). Microbleeds and hemorrhages were not observed from the C57VCI mice. Out of the 17 C57VCI mice, infarcts were detected from a total of six mice (C57VCI 4, 5, 9, 15, 16, and 17) (Table S1). A variation in size and appearance of cerebral infarcts was, however, noted from the MR images (Figure 1). The appearance and size of the infarcts differed among the C57VCI mice. For example, infarcts were first observed at Day 15 for mouse C57VCI 4, Day 29 for C57VCI 5, and Day 8 for C57VCI 9 (Figure 1A). Interestingly, there was one mouse that displayed infarcts in both hemispheres (C57VCI 17) and the septal nuclei (Figure 1A). For this mouse, infarcts were first observed and detected only in the right hemisphere at day 22 but when images were taken subsequently at day 29, infarcts were also observed from the opposite hemisphere (left) and the septal nuclei (Figure 1A).

**Figure 2:**
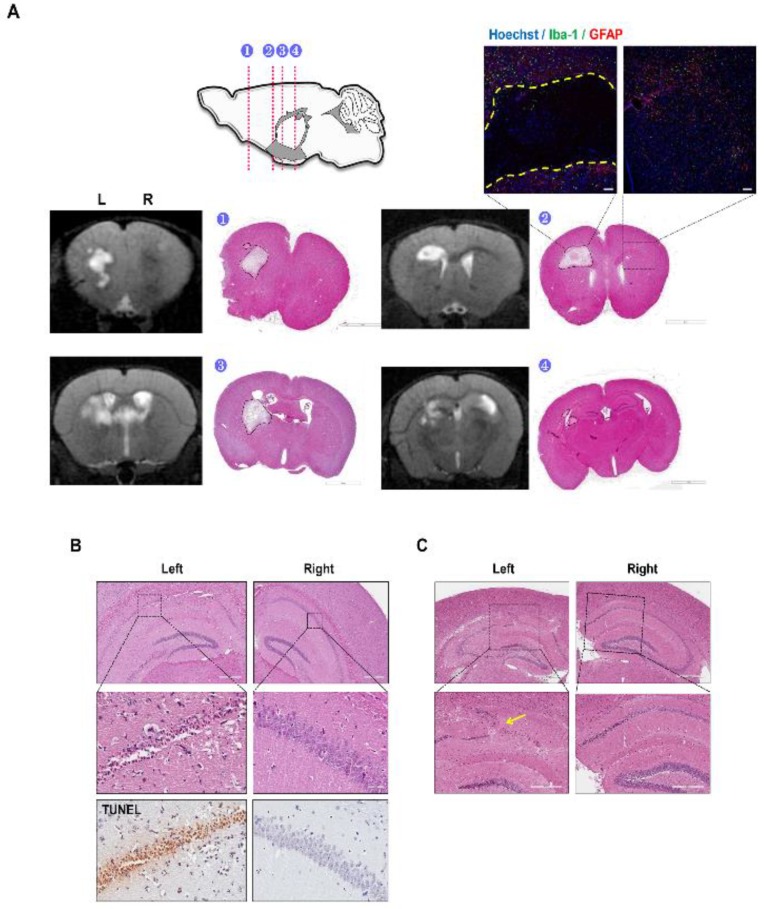
Pathological features of C57VCI mice. Infarcts observed from the T2 weighted MR images were confirmed through histological staining (H&E). (1) forceps minor, (2) external capsule of the corpus callosum, (3) internal capsule and caudate putamen, and (4) hippocampal fimbria. (**A**) Immunohistochemical staining was performed to observe expressions of microglia (Iba-1) and astrocytes (GFAP) in the contralesional (upper inset: right) and ipsilesional sides (upper inset: left). L indicates left and R indicates the right hemisphere of the mouse brain. Scale bar = 2 mm (H&E), 10 μm (Confocal image, upper right inset). (**B**) Loss of neuronal cells in the unilateral (left side in this case) hippocampus was observed by H&E and TUNEL stains. (**C**) Solid yellow arrow indicates abnormal folding of the hippocampal structure in the left hippocampus. Scale bars = (**B**) From top to bottom: 400, 50, and 50 μm, (**C**) From top to bottom: 600 and 300 μm.

A total of 18 infarcts were detected from the six mice and many of the infarcts were observed from the right hemisphere (on the ameroid side) (Figure 1B). Many of the cerebral infarcts were detected in the following areas of the mouse brain: caudate putamen (CPu), corpus callosum (CC), anterior commissure (AC), cortex, and hippocampus (hippo). The highest number of infarcts were observed from the right CC followed by the right and left CPu, respectively (Figure 1C). The volume of the cerebral infarcts ranged from 0.051 to 4.774 mm^3^ (Figure 1D). The infarct with the greatest volume was detected in the left CC (Figure 1D). Cerebral infarcts visualized from the MR images were corroborated by H&E staining (Figure 2A). The MR images and the corresponding H&E stains were visualized from four representative areas: forceps minor of CC (indicated as 1), external capsule of CC (indicated as 2), internal capsule and caudate putamen (indicated as 3), hippocampal fimbria (indicated as 4), and or the cerebral cortex (not noted in Figure 2A). When immunostaining was performed, the infarct site was easily demarcated (black broken line; Figure 2A) as an empty void and signs of inflammatory cell infiltration (Iba-1 and GFAP) were not observed. 

### 2.3. Decreased Hippocampal Neuronal Density and Increased Iba-1 and GFAP Immunoreactivies Were Observed from the C57VCI Mice

Along with cerebral infarcts, C57VCI mice also showed selective neuronal death in the CA1 and CA2 areas of the hippocampus (Figure 2B). The lesion was usually unilateral (out of the six C57VCI mice that survived up to Day 32, three showed loss on the ameroid side and one on the microcoil side) and compared to the contralesional hippocampus, pyknosis and TUNEL positive cells were detected from the ipsilesional pyramidal neurons (Figure 2B). Along with hippocampal neuronal death, alterations in the hippocampal structure such as hippocampal folding (indicated by a yellow arrow) were also evident on the ipsilesional side in a few mice (Figure 2C).

**Figure 3:**
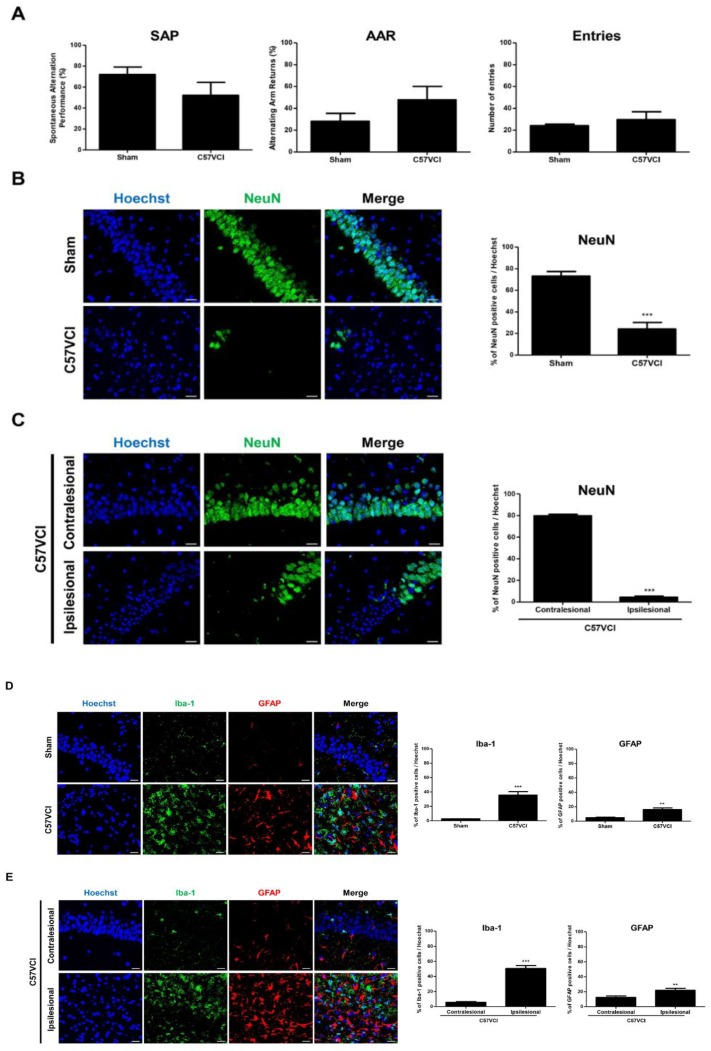
Changes in spatial working memory, neuronal density, and microglial/astrocyte activity following VCI surgery. (**A**) Changes in spatial working memory were assessed using the Y-maze on Day 32 after VCI was induced. Behavioral indices from left to right: spontaneous alternation performance (SAP), alternating arm returns (AAR), and number of entries. (**B**,**C**) NeuN immunostaining (green) of the hippocampus. (**B**) *** *p* < 0.001 vs. sham; mean ± S.E.M. (**C**) *** *p* < 0.001 vs. contralesional; mean ± S.E.M. Scale bar = 20 μm. (**D**,**E**) Sham and C57VCI mouse brain sections were double immunostained with antibodies to Iba-1 (green), a microglial marker, and GFAP (red), an astrocyte marker. (**D**) ** *p* < 0.01, *** *p* < 0.001 vs. sham; mean ± S.E.M. (**E**) The contralesional side of the C57VCI group was used as a control instead of the sham group. ** *p* < 0.01, *** *p* < 0.001 vs. contralesional; mean ± S.E.M. Scale bar = 20 μm.

The three sham mice and six C57VCI mice that survived up to 32 days (C57VCI 1, 2, 5, 6, 11, and 12) following VCI surgery were subjected to the Y maze test to assess changes in spatial working memory. Compared to the sham group, a drop in spontaneous alternation performance (SAP) % was evident from the C57VCI group (sham: 71.8% ± 7.3%, C57VCI: 52.2% ± 12.3%; Figure 3A). However, the difference was not significant. Furthermore, statistically significant differences in the alternating arm return % (AAR) and the total number of entries were not noted between the groups. 

Immunohistochemical staining was carried out to further assess hippocampal neuronal density in the sham and C57VCI groups by using the mature neuron marker, NeuN [19]. As observed from the H&E stains, compared to the sham group (73.2% ± 4.2%), a statistically significant decrease in NeuN positive neuronal cell density detected from the hippocampi of the C57VCI group (24.1% ± 6.2%, *** *p* < 0.001; Figure 3B). To further investigate on the unilateral damage of VCI-induced mice, four of the four C57VCI mice (C57VCI 2, 5, 6, and 11) that displayed hippocampal lesions were analyzed separately. Differences in NeuN positive neuronal cell density were accentuated when the ipsilesional (4.4% ± 0.9%, *** *p* < 0.001) side was compared to the contralesional (79.8% ± 1.4%) side (Figure 3C). Additional IHC was carried out to assess proliferation of activated microglia (Iba-1) and astrocytes (GFAP) at the hippocampal regions. The expression levels of Iba-1 microglia positive and GFAP astrocyte positive cells in the hippocampi of sham and C57VCI groups were as follows, Iba-1: sham (2.6% ± 0.2%) and C57VCI (35.6% ± 4.9%, *** *p* < 0.001), GFAP: sham (4.8% ± 0.8%) and C57VCI (16.1% ± 2.4%, ** *p* < 0.01). Compared to the sham group, a 13.7 and 3.4-fold increase (statistically significant) in expressions of Iba-1 and GFAP positive cells, respectively, was observed from the C57VCI group (Figure 3D). Iba-1 and GFAP expression levels were also assessed by comparing the contralesional and ipsilesional sides of the four (out of six) C57VCI mice that displayed hippocampal lesions. Iba-1 and GFAP expression levels were as follows, Iba-1: contralesional (5.7% ± 0.8%) and ipsilesional (50.7% ± 3.8%, *** *p* <0.001), GFAP: contralesional (12.5% ± 1.9%) and ipsilesional (22.0% ± 2.7%, ** *p* < 0.01) (Figure 3E). 

### 2.4. Differences in DTI Parameters Were Not Significant without the Presence of Infarcts

**Figure 4:**
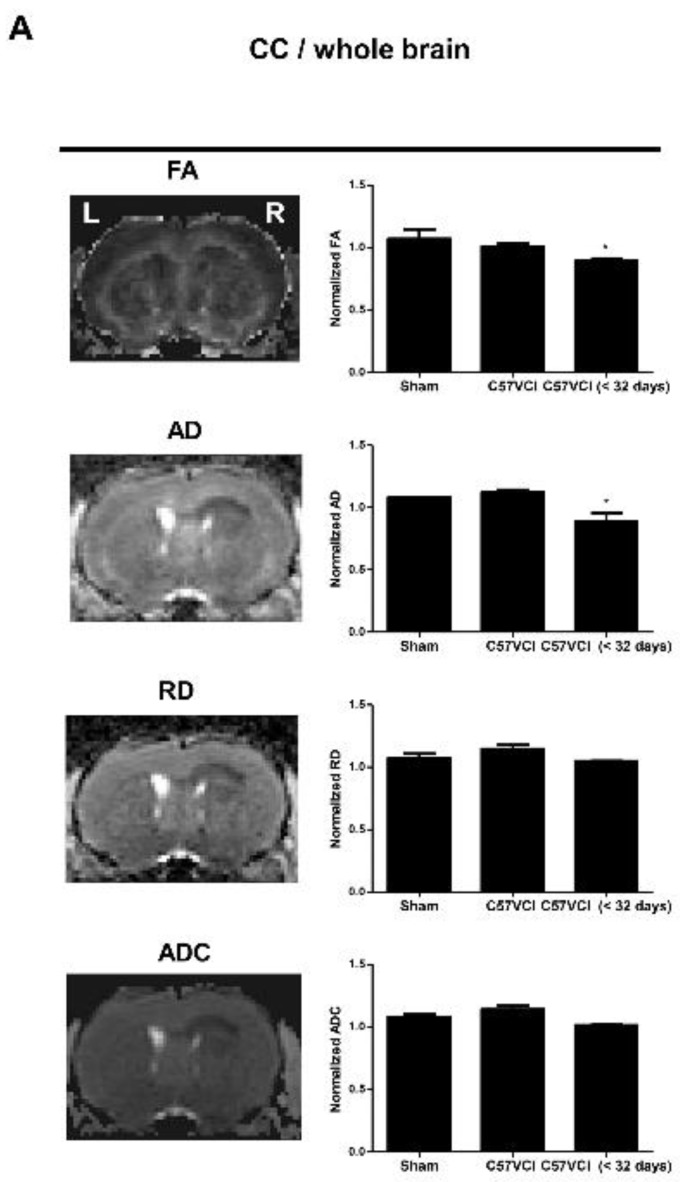

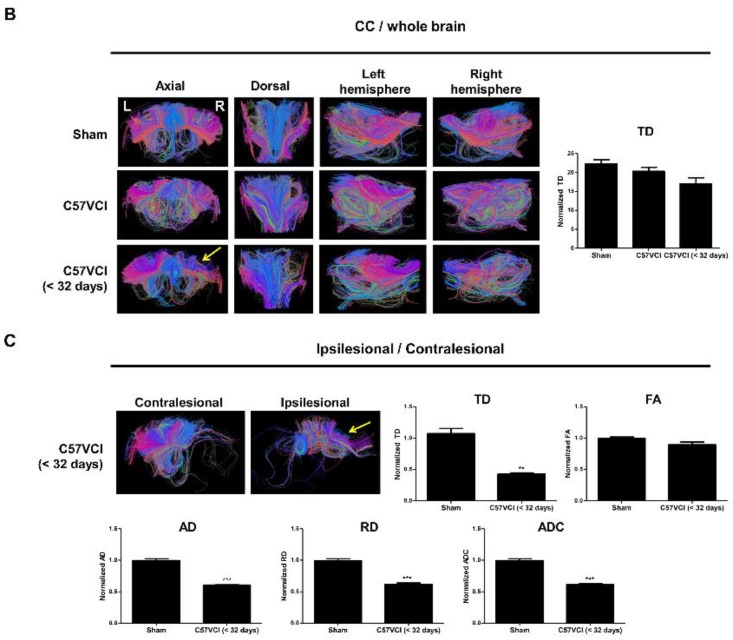
The effects of VCI on Diffusion Tensor Imaging (DTI) parameters. (**A**) Tract Density (TD), Axial Diffusivity (AD), Radial Diffusivity (RD), and Apparent Diffusion Coefficient (ADC) maps of the corpus callosum (CC) from a representative animal (far left). The normalized values of the CC (the CC value were divided by the value of the whole brain) were used for group comparisons: Sham, C57VCI (sacrificed at 32 days), and C57VCI <32 days are shown in bar graphs on the right. * *p* < 0.05 vs. sham; mean ± S.E.M. L indicates left and R indicates the right hemisphere of the mouse brain. (**B**) Tractography of a representative animal from each of the groups (from left to right: axial, dorsal, left hemisphere, right hemisphere) and the normalized (CC/whole brain) tract density shown as bar graphs. The solid yellow arrows indicate reduced fiber density. (**C**) Normalized DTI parameters (the value on the side of the infarct was divided by the value of the contralesional side of the C57VCI <32 days group) quantitated by comparing the ipsilesional to the contralesional side of the C57VCI <32 days group. ** *p* < 0.01, *** *p* < 0.001 vs. contralesional; mean ± S.E.M.

Interestingly, when comparing the C57VCI (six that survived up to 32 days; C57VCI 1,2,5,6,11, and 12) to the sham group, statistically significant differences in DTI indices (normalized = entire corpus callosum (CC)/whole brain) were not observed (Figure 4A). Although tract density was slightly reduced (normalized: CC/whole brain), the difference was not statistically significant (Figure 4B). We further investigated whether the presence of infarcts in the CC affect DTI parameters (normalized: entire CC including infarct/whole brain). 

Three C57VCI mice that died before the 32 days with cerebral infarcts (C57VCI <32 days: C57VCI 15, 16, and 17) were included in the analyses. Compared to the sham group, a statistically significant reduction in FA (* *p* < 0.05) and AD (* *p* < 0.05) were observed from the C57VCI <32 days group (Figure 4A). Interestingly, significant changes in demyelination (RD) and tissue integrity (MD) were not observed. When observing the tractography of the corpus callosum, reduced fiber density (yellow solid arrow; Figure 4B) was noted on the ipsilesional side of the C57VCI <32 days group. However, when compared to the tract density of the sham group, differences were not significant. Instead of measuring the DTI indices of the entire corpus callosum, DTI indices of the contralesional side were also compared to that of the ipsilesional side (Figure 4C). Pronounced effects on the DTI parameters were noted when excluding the sham group and comparing the contralesional to the ipsilesional side (Figure 4C). Compared to the contralesional side, a significant reduction in TD (** *p* < 0.01), AD (*** *p* < 0.001), RD (*** *p* < 0.001), and ADC (*** *p* < 0.001) were noted in the ipsilesional side.

### 2.5. 5XVCI Mice Displayed Pathological Heterogeneity and Reduced Aβ Levels in the Hippocampus

**Figure 5:**
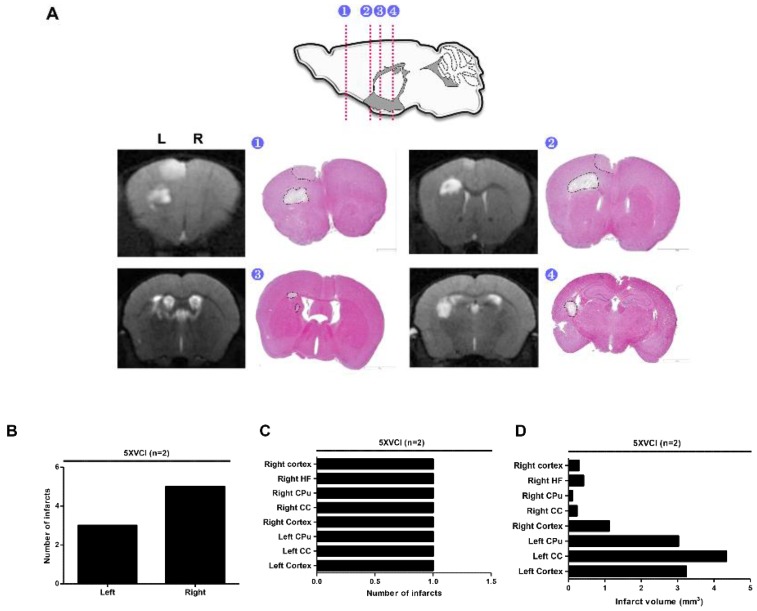

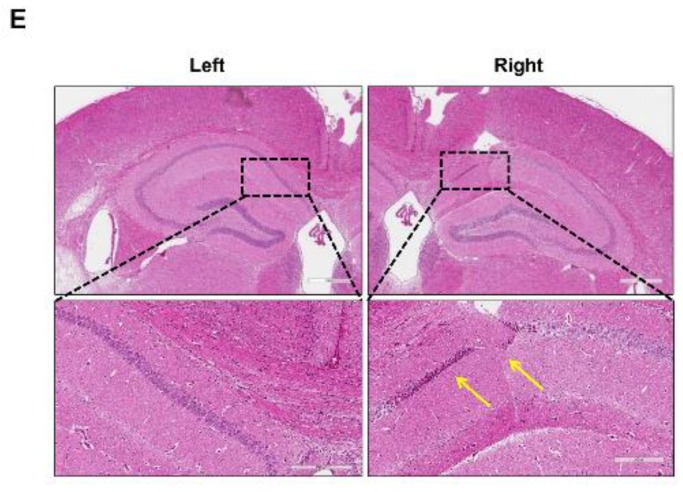
Pathological features of 5XVCI mice. (**A**) Locations of the infarcts were corroborated by matching MR images with the respective histological stains. Compared to WT VCI mice, infarcts appeared in similar locations. L indicates left and R indicates the right hemisphere of the mouse brain. (1) forceps minor (2) external capsule of the corpus callosum (3) caudate putamen (4) hippocampal fimbria. (**B**) Total number of infarcts observed from 2 of the 5 5XVCI mice (5XVCI 5, 6). (**C**) Distribution of infarcts. CC = corpus callosum, CPu = caudate putamen, AC = anterior commissure, Hippo = hippocampus, and HF = hippocampal fimbria. (**D**) Volume of the cerebral infarcts (mm^3^). (**E**) Hippocampal neuronal death (left solid yellow arrow) and structural alterations (right solid yellow arrow) in the hippocampus were observed in the unilateral hippocampus of 5XVCI mice. Scale bar = (**A**) 2 mm, (**E**) From top to bottom: 400, 200 μm.

Following the experiment using C57VCI mice, a second experiment was performed to assess whether it would be feasible to detect changes in AD pathogenesis by 5XFAD AD mice to VCI (5XVCI) (Figure S2). Interestingly, the survival rate of the 5XFAD mice was strikingly higher (83.3%) when compared to that of the C57VCI mice (Figure S2). Out of a total of six 5XVCI mice, only one mouse died at Day 22 (5XVCI 5) while the remaining five mice survived up to Day 32 (5XVCI 1,2,3,4, and 6) (Figure S2). Only two out of the six 5XVCI mice (5XVCI 5 and 6) exhibited infarcts in the parenchyma (Table S2). Like the C57VCI mice, 5XVCI mice also exhibited signs of cerebral infarcts in regions such as the corpus callosum (CC), caudate putamen (CPu), and hippocampal fimbria (HF) (Figure 5A). Most of the infarcts were observed from the right hemisphere of the 5XVCI mice (Figure 5B,C). The volume of the cerebral infarcts ranged from 0.102 to 4.339 mm^3^ (Figure 5D). Like the C57VCI group, the infarct with the greatest volume was detected in the left CC of the 5XVCI group (Figure 5D). 

**Figure 6:**
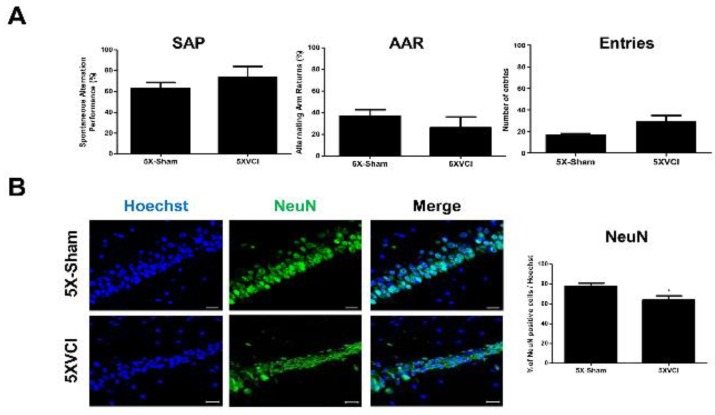

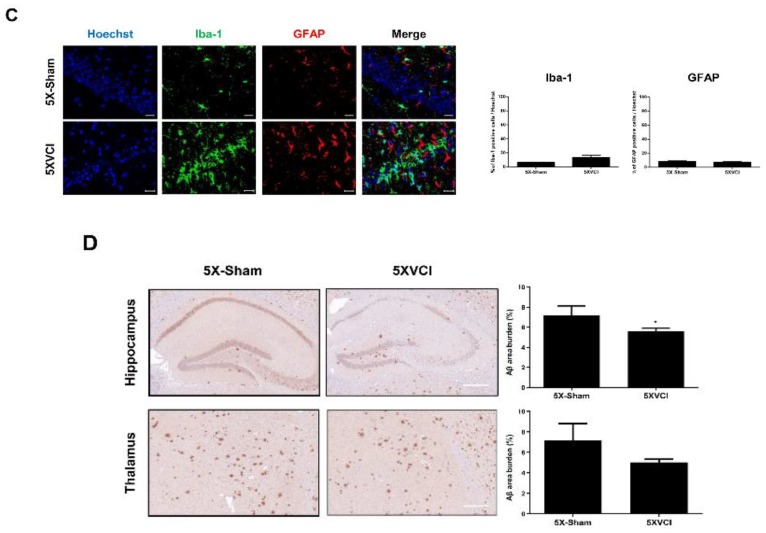
Assessment of changes in neuronal density, microglial/astrocyte activity, and amyloid burden of 5XVCI mice. (**A**) According to the Y-maze results, an impairment in spatial working memory was not observed from the 5XVCI mice. (**B**)According to NeuN immunostaining (green), a significant reduction in hippocampal neuronal density was observed from the 5XVCI group. * *p* < 0.05 vs.5X-Sham; mean ± S.E.M, scale bar = 20 μm. (**C**) Significant changes in microglia and astrocyte activities were not observed from the double immunostaining of Iba-1 (green) and GFAP (red) markers, respectively. Mean ± S.E.M, scale bar = 20 μm. (**D**) There was a significant reduction in 6E10 immunoreactivity in the hippocampi of 5XVCI mice compared to the 5X-Sham mice. * *p* <0.05 vs.5X-Sham; mean ± S.E.M, scale bar = 200 μm.

Hippocampal neuronal death was also observed unilaterally in the CA1 and CA2 regions of the right hippocampus (yellow arrow; Figure 5E). Hippocampal folding was also discernible (Figure 5E). Like the C57VCI mice, a heterogeneity was noted in the manifestation of pathological features (presence of infarcts and neuronal loss) in 5XVCI mice (Table S2). Only two of the five 5XVCI mice that survived up to 32 days (5XVCI 1 and 4) showed signs of hippocampal neuronal loss (Table S2). A slight reduction in NeuN positive neuronal cell density was noted but impairment in spatial working memory (SAP) was not observed from the 5XVCI mice (Figure 6A,B). In comparison to the 5X-Sham group (63.0% ± 5.8%) the SAP % was higher for the 5XVCI group (73.9% ± 10.1%). Moreover, statistically significant differences in AAR% and number of entries were also not noted (Figure 6A). NeuN neuronal density levels were as follows: 5X-Sham: 77.7% ± 3.0%, 5XVCI: 63.9% ± 4.3%, * *p* < 0.05) (Figure 6B). Although the fold change was not as high as the C57VCI mice, a slight increase in Iba-1 microglia expression was observed from the hippocampal region of the 5XVCI group (13.4% ± 3.2%) in comparison to the 5X-Sham group (6.2% ± 0.7%). Dramatic differences in GFAP positive astrocyte expressions were not noted between the two groups: 5X-Sham: 8.2% ± 0.7%, 5XVCI: 6.8% ± 0.9% (Figure 6C).

Aβ IHC staining was carried out to evaluate changes in amyloid burden of 5XFAD after inducing VCI. The 6E10 Aβ monoclonal antibody was used to stain all Aβ expressing cells and plaques in the hippocampi and thalamus of both 5X-Sham and 5XVCI mice. The area of amyloid burden was quantitated afterwards. While no significant changes were observed from the thalamus (5X-Sham: 7.2% ± 1.7%, 5XVCI: 5.0% ± 0.3%, * *p* < 0.05), a significant reduction in area of amyloid burden in the hippocampus was noted from the 5XVCI group (Figure 6D; 5X-Sham: 7.2% ± 0.9%, 5XVCI: 5.6% ± 0.3%). 

## 3. Discussion

In the present study, we observed the heterogeneous disease progression and inconsistent reproducibility of both WT C57BL6/J and transgenic AD 5XFAD mice subjected to asymmetric vascular compromise. One finding that was noticeable in our study was that the mortality rate of the C57VCI mice was strikingly high. In comparison to a previously reported study [7] where the survival rate of the VCI-induced mice was around 80% at post 28 days, the survival rate for the current study was around 27%. It has been reported in the past that the C57BL/6 strain is highly susceptible to cerebral ischemia when compared to other strains such as the ICR, BALB/c, and C3H strains [20]. Previous studies also showed that severe ischemia occurs in the C57BL/6 strain when bilateral CCA occlusion was performed by temporarily occluding the carotid artery using microaneurysm clips for 20 minutes [20]. The poorly developed or even absent posterior communicating artery in close to 90% of the C57BL6/J mice [8,21] could have accounted for this high susceptibility. Since the C57BL/6 strain is more prone to developing cerebral infarcts, it makes it difficult to control the onset, size, and number of infarcts that will develop in the mouse brain. This could partly explain the pathological heterogeneity observed specifically in relation to the presence and severity of cerebral infarcts observed from the VCI-induced mice of the current study. It is interesting to note that a unilateral application of a single ameroid constrictor (inner diameter of 0.5 mm with a microcoil applied around the opposite CCA) was able to exert effects of similar magnitude (high mortality rate and multiple cerebral infarcts) comparative to models where ameroid constrictors were applied bilaterally to the CCAs [8].

Previously reported [7] pathological features such as cerebral infarcts and hippocampal neuronal loss were reproduced from both C57VCI and 5XVCI mice. However, there was a wide variation in terms of which hemisphere the infarcts appeared at. Out of the six C57VCI mice that survived up to 32 days, only one of the mice (C57VCI 5) displayed signs of infarct that was on the left or microcoil side. Out of the entire 17 C57VCI mice (including mice that died before 32 days), one displayed infarcts on the microcoil side (L), three on the ameroid side (R), and one on both hemispheres. Such results contrasted strongly to past findings where 81% of VCI-induced mice (day 32) displayed multiple infarcts in the right hemisphere [7]. Similarly, out of the six 5XVCI (including one mouse that died before 32 days) mice, one mouse exhibited infarcts on the microcoil side (L) side while infarcts were detected on the ameroid side (R) of another mouse. With respect to anatomical localization, the highest number of infarcts were detected from the right CC of C57VCI mice while an even distribution of infarcts was observed from the 5XVCI mice. Interestingly, the greatest infarct volumes were observed from the left CC for both groups. Such results are not surprising in that the CC is part of the watershed territory which makes it a highly susceptible region to ischemia. 

Like subcortical infarcts, it was expected that hippocampal neuronal loss would be predominantly observed from the ameroid side. However, out of the six C57VCI mice that survived up to 32 days, only three mice showed signs of hippocampal neuronal death in the ameroid side (R) and one mouse on the microcoil side (L). Out of the entire 17 C57VCI mice, when including mice that died before 32 days, there were two other mice that showed signs of neuronal loss in the microcoil side. Hippocampal neuronal death for the 5XVCI mice was discernible from three mice (including one mouse that died before 32 days) in the ameroid side (R), while unlike the C57VCI mice, no signs of neuronal loss were visible from the microcoil side (L) from the other remaining mice. A noticeable feature is that previously 69% of the VCI-induced mice (day 32) showed signs of hippocampal neuronal death on the ameroid side [7] while for the current study it was 50% (three of the three C57VCI mice that survived up to 32 days) and 40% (two of the five 5XVCI mice that survived up to 32 days) for the C57VCI and 5XVCI groups, respectively. 

It is known that hypoperfusion can lead to border zone infarcts and hippocampal sclerosis [22]. In accordance with past findings, increased levels of Iba-1positive microglia and GFAP positive astrocytes were observed from the hippocampi of the C57VCI group. An increase in microglia number is known to be a noticeable feature of hypoperfusion models [6,23,24]. Microglia is reported to be indicative of the severity of ischemic injury [25]. Along with microglia, levels of GFAP which is a marker used to label reactive astrocytes [26], is also increased in chronic hypoperfusion models [27]. A reduction in NeuN neuronal density and presence of apoptotic neurons in the CA1 and two hippocampal regions of our C57VCI mice was observed via IHC and TUNEL staining, respectively. This increase in cell apoptosis could have accounted for the activation of the astrocytes and microglial cells at the site of hippocampal lesion [28] considering that both astrocytes and microglia cells have been reported to be involved in the clearance of apoptotic neurons [29]. 

A past study reported that bilateral hippocampal lesions impair the spatial working memory of a rat model [30] while another group presented findings that large dorsal hippocampal lesions which encompass 40%–60% of the total hippocampal volume impair the spatial working memory of rats [31]. Smaller lesions, however, preserved the memory. In the current study, neuronal loss was evident from the unilateral hippocampus for both the C57VCI and 5XVCI mice, but the loss involved only the CA1 and 2 regions, not the entire hippocampus. Since the extent of hippocampal damage was not widespread, partial lesions might not have been enough to further impair the spatial working memory of the VCI groups. Furthermore, Y-maze was conducted at Day 32, while only three of the six C57VCI and two of the five 5XVCI mice that survived up to Day 32 showed signs of neuronal loss. Thus, there were more mice with an intact hippocampus and preserved spatial working memory remaining in each of the groups. This would have factored in increasing instead of decreasing the overall SAP of both the C57VCI and 5XVCI groups.

Diffusion tensor imaging (DTI) is known to be sensitive towards the motion of water molecules. One of the parameters of DTI is fractional anisotropy (FA). A decrease in FA indicates decrease in anisotropic water diffusion (direction-dependent) which is indicative of damage to the white matter region [32,33]. Axonal degradation can be measured by axial diffusivity (AD), demyelination can be assessed by measuring radial diffusivity (RD), and tissue integrity can also be assessed by measuring apparent diffusion coefficient (ADC) [33]. Tract density (TD) was quantitated to determine whether the presence of lesions also affects the number and the density of the tracts of the corpus callosum. 

In this present study, signs of white matter damage, specifically in the corpus callosum, were assessed non-invasively by utilizing DTI. We expected that white matter rarefaction would occur predominantly on the left hemisphere (microcoil side), which, however, could not be determined due to the heterogeneous pathology of the C57VCI mice. Infarcts and hippocampal neuronal loss were observed randomly in both hemispheres and thus it was difficult to isolate the effects of ameroid constrictors from that of microcoils. Out of the six C57VCI mice that survived up to 32 days, only one mouse displayed infarcts in the microcoil side. For that mouse, infarcts were not detected in the corpus callosum. The overall lack in presence of infarcts could have accounted for the insignificant difference observed between the sham and C57VCI group (all four DTI indices; CC/whole brain). When the three C57VCI mice that died before the 32 days with cerebral infarcts (C57VCI<32 days: C57VCI 15, 16, and 17) were compared to the sham group, a reduction in FA and AD values was evident. The results suggest that the presence of infarcts in the corpus callosum increased both white matter damage (FA) and axonal degeneration (AD). Moreover, past groups have reported that a decrease in FA and AD values is indicative of early stage white matter degeneration [34,35]. 

Various studies have reported on how it is common to observe the co-occurrence of Alzheimer’s disease and vascular pathology [36,37,38] in human patients. However, few studies have investigated on creating an AD-VCI mouse model. To the best of our knowledge, this is the first study to induce VCI in the 5XFAD transgenic AD mouse model and to study the resulting effects. The 5XFAD mouse model is a well-characterized, suitable model to study AD amyloid pathology [39]. An interesting observation made from our 5XVCI experiment was the reduction in Aβ area in the hippocampus. These results were similar to a previous report, where subjecting mice overexpressing the APP Swedish and Indiana mutation to BCAS reduced Aβ deposition and cored plaque (insoluble Aβ) formation but expedited neuronal loss and memory impairment [40]. The authors proposed that chronic hypoperfusion could have led to a reduction in insoluble Aβ via increased inflammation or shift in Aβ solubility [40]. However, the effects of chronic hypoperfusion in AD pathology is debatable. One group has noted an increase in Aβ deposition, a month after BCAS was induced, in a transgenic AD mouse model with Swedish, Dutch, and Iowa mutations [41]. Furthermore, an increase in total number of Aβ plaques was also demonstrated in a mouse of model of severe chronic cerebral hypoperfusion (SCCH), which was induced by ligating the right CCA with a silk thread and applying a vessel clamp to the left CCA of APPswe/PS1 mice [13]. 

The selective susceptibility of the hippocampus towards ischemic brain injury [42] could partly explain why changes in amyloid deposition of the 5XVCI mice were only distinct in the hippocampus and not the thalamus. The common carotid artery branches out into many arteries including the middle cerebral arteries (MCA) and the posterior cerebral arteries (PCA) [43]. The PCA plays a role in hippocampal formation by providing blood supply to the hippocampal region [44]. Thus, although only two of the five 5XVCI mice displayed cerebral infarcts, all of the 5XVCI mice would have undergone stenosis of the CCA over the course of 32 days which would have affected the blood flow and the dynamics of amyloid deposition in the hippocampus. Furthermore, the 6E10 antibody used to evaluate amyloid deposition is reactive towards Aβ proteins which are expressed not only in plaques but also in the neuronal cells of the hippocampus. Since neuronal loss was evident from two of the five 5XVCI mice, this could have factored in reducing the overall level of amyloid burden (in the hippocampus) of the 5XVCI group. It has been reported in the past that at injured or affected areas of the brain, there is an intense expression of activated microglia while amyloid deposition is absent [45]. This may be consistent with our results that the 5XVCI group which exhibited decreased levels of amyloid burden in the hippocampus showed, although not statistically significant, a 2.2-fold increase in Iba-1 positive microglia expression in comparison to the 5X-Sham group. 

Limitations of this study included small sample size and absence of a baseline. The heterogeneous disease progression observed from this current study underscore the need of setting a baseline. Temporal profiles of the cerebral blood flow (CBF) were not recorded. Measuring the hemodynamics at baseline and at different time points could have provided the means of making a criterion to exclude certain mice subjected to VCI below a set cut-off. Such exclusions could have reduced heterogeneity and improved the overall reproducibility of the model in this study.

Taken together, the pathological heterogeneity observed from the current study recapitulates the heterogeneous nature of VCI that is observed clinically. The complicated progressions of two different pathologies (AD and vascular) could attribute to differences in disease progression that were observed between the C57VCI and 5XVCI mice. Further research is warranted to elucidate this difference and the complex mechanisms underlying asymmetric vascular compromise. Such investigations may provide solutions to increase the reproducibility of mouse VCI models which will be essential for the model to be used as a platform to test novel treatments for human VCI.

## 4. Materials and Methods

### 4.1. Ethical Statement

This research was approved by the Institutional Animal Care and Use Committee (IACUC) of the Samsung Biomedical Research Institute (SBRI) at Samsung Medical Center (SMC). As an accredited facility of the Association for Assessment and Accreditation of Laboratory Animal Care International (AAALAC International), SBRI abides by the Institute of Laboratory Animal Resources (ILAR) guide.

### 4.2. Mice

This study consisted of a total of 20 C57BL6/J (female, 3-4-month-old, Sham: *n* = 3, C57VCI: *n* = 17) and 8 transgenic AD 5XFAD (female, 3–4 month old; 5X-Sham: *n* = 2, 5XVCI: *n* = 6) mice. Both C57BL6/J and 5XFAD were originally purchased from the Jackson Laboratory (Bar Harbor, ME, USA). Both strains were maintained by mating a 5XFAD male with a C57BL6/J female mouse. Genotyping was performed from mouse tail biopsies of the offspring and the transgenic and non-transgenic littermates were separated accordingly. Mice were maintained in a 12-hour light/12-hour dark cycle and were fed ad libitum.

### 4.3. VCI Modeling

Initially, anesthesia was induced by placing the mice in a closed induction chamber. The induction setting was 5% isoflurane (Hana Pharmaceutical Co., Ltd., Seoul, Republic of Korea). Mice were then placed in a supine position and anesthesia was maintained at 1.5~2% during the surgical procedure. VCI was induced (Figure 1) by referring to previously reported studies [7,46]. Once the fur was removed and the incision area was sterilized with povidone iodine, a paramedian skin incision was performed right above the thyroid bone. Then, the omohyoid and sternomastoid muscles were retracted. Both the left and right carotid arteries were exposed by detaching the carotid arteries from the overlying fascia and surrounding sheath including the vagus nerve. An ameroid constrictor with a titanium jacket, an inner diameter of 0.5 mm, outer diameter of 3.25 mm, and length of 1.28 mm (Research Instruments SW, Escondido, CA, USA) was placed around the right common carotid artery (CCA) by lifting the distal and proximal ends of the CCA by using a 6-0 silk thread (Ethicon, Cincinnati, OH, USA). Surrounded by a circular, ring-shaped titanium jacket, the ameroid constrictors were applied to the CCAs through an opening slit. With the same procedure carried out on the left CCA, once the artery was exposed, a microcoil (Wuxi Samini Spring Co., Ltd., Wuxi, China) with an inner diameter of 0.18 mm was implanted by rotating the coil around the CCA right below the bifurcation point. The sham operated animals underwent the same procedure as the C57VCI group but no microcoil or ameroid constrictors were applied after exposing the bilateral CCAs. After the surgical procedure, the midline incision was closed by suturing the area with a 5-0 suture silk thread (Ethicon, Cincinnati, OH, USA). Mice that have undergone VCI surgery were sacrificed at Day 32. A Kaplan-Meier survival curve was drawn using the GraphPad Prism software to assess survival rates. 

### 4.4. Magnetic Resonance Imaging and Data Processing

MR images were acquired on a 7T/20 MR System (Bruker-Biospin, Ettlingen, Germany) equipped with a 20 cm gradient set capable of supplying up to 400 mT/m in 100 µs rise-time. A quadrature birdcage coil (inner diameter of 72 mm; Bruker-Biospin) was used for excitation, and an actively decoupled phased array brain coil was used for signal reception. All mice were anesthetized under 2% isoflurane while acquiring the MR images. A T2 weighted spin echo sequence was used to acquire MR images. The parameters were as follows: repetition time (TR)/echo time (TE) = 3000/60 ms, number of averages = 6, echo train length = 4; in-plane resolution = 100 × 100 μm^2^; slice thickness = 0.5mm. A diffusion weighted spin echo was used to carry out Diffusion Tractography Imaging (DTI). The parameters were as follows: TR/TE = 2000/30 ms, number of average = 4, field of view (FOV) = 20 × 15 mm^2^, matrix = 128 × 96, in-plane resolution = 156 × 156 μm^2^, slice thickness = 0.5 mm, number of slices = 16, gradient direction = 30, diffusion gradient duration = 4.5 ms, diffusion gradient separations = 10.6 ms, and b-values = 1000 s/mm^2^.

After the DTI images were acquired, the FSL package was utilized to perform head motion and eddy-current distortions. Once the motion control was performed, the Diffusion Toolkit and TrackVis software (www.trackvis.org) were utilized to draw separate region of interests (ROIs) around the corpus callosum and the whole brain. Both the medial and lateral regions (both hemispheres) of the corpus callosum were included when drawing the ROI. The mean fractional anisotropy (FA), radial diffusivity (RD), apparent diffusion coefficient (ADC), and tract density (TD) were quantitated from the ROIs. Tract density was calculated by dividing the number of tracts by the voxel number of the respective ROI (whole brain or corpus callosum). The TD/FA/AD/RD/ADC of the corpus callosum was divided by the TD/FA/AD/RD/ADC of the whole brain, respectively to obtain normalized values. Three C57VCI mice (C57VCI 15,16, and17) that died before the 32-day endpoint (C57VCI < 32 days) and had cerebral infarcts were also separately analyzed. The TD/FA/AD/RD/ADC of the infarct side of the CC (ipsilesional) was divided by the TD/FA/AD/RD/ADC of the contralesional CC, respectively. The MRICro (www.mricro.com) software was also used to measure the infarct volume of the VCI induced mice. 

### 4.5. Histological Staining and Analyses

All sham and VCI-operated mice (both C57VCI and 5XVCI) were sacrificed through cardiac perfusion 32 days after VCI was induced. Post-mortem examination of several mice that died before 32 days was also performed. Brain tissues were harvested and fixated in 4% paraformaldehyde (Biosesang, Republic of Korea) for a day before paraffin blocks were made. Sections of 4 μm were made of the paraffin brain blocks using a micrometer (Leica Biosystems, Wetzlar, Germany). Slides were deparaffinized with xylene and varying percentages of ethanol. 1X Citrate buffer (pH 6; Dako, Carpinteria, CA, USA) was then used to perform heat antigen retrieval. Prior to performing immunohistochemical (IHC) staining, Hematoxylin and Eosin (H&E; Dako, CA, USA) was performed to examine changes in histology such as the presence of cerebral infarcts or neuronal loss following VCI surgery. Terminal deoxynucleotidyl transferase dUTP nick end labeling (TUNEL) staining (Millipore, Temecula, CA, USA) was performed according to the manufacturer’s instructions.

Immunofluorescence staining was carried out as reported previously [47] using primary antibodies against ionized calcium binding adaptor molecule 1 (Iba-1, 1:250; Wako Chemicals, Richmond, VA, USA), glial fibrillary acidic protein (GFAP, 1:1000; Abcam, Cambridge, MA, USA), and neuronal nuclear antigen (NeuN, 1:400, Millipore). Alexa Fluor 488-conjugated donkey anti-mouse (1:400; Life Technologies, Hudson, NH, USA), Alexa Fluor 546-conjugated goat anti-rabbit (1:400; Life Technologies, Hudson, NH, USA), and Alexa Fluor 647-conjugated donkey anti-goat (1:400; Life Technologies, USA) were used as secondary antibodies. Iba-1, GFAP, and NeuN are markers that are commonly used to identify microglia, astrocytes, and mature neurons, respectively. NeuN was used to examine changes in the neuronal density of the hippocampi and Iba-1 and GFAP were used to evaluate the proliferation of inflammatory cells at sites of neuronal loss. 3,3’-Diaminobenzidine (DAB) staining was performed as reported previously [48] for the primary antibody against amino acid residue 1-16 of beta amyloid (Aβ) (6E10; 1:250; Biolegend, San Diego, CA, USA). This antibody was used to detect changes in amyloid deposition after subjecting 5XFAD mice to VCI surgery. 

All H&E and DAB stained slides were scanned using the Scanscope AT scanner (Aperio Technologies, Vista, CA, USA). Images of slides that underwent immunofluorescent staining were acquired using a confocal microscope (Carl Zeiss AG, Jena, Germany). The total number of Iba-1, or GFAP, or NeuN negative and positive cells were manually counted for each image by using the ImageJ image processing program (National Institutes of Health (NIH)). DAB stained slides were also scanned using the Vectra® Automated Imaging System (PerkinElmer Applied Biosystems, Waltham, MA, USA) where a spectral un-mixing algorithm quantitated each of the spectral components (DAB and Hematoxylin). The InForm 2.4.1 image analysis software was used to quantitate the area percentage of Aβ burden in the hippocampi and thalamus of 5X-Sham and 5XVCI mice. 

### 4.6. Assessment of Changes in Behavioral Performance following VCI Surgery

The Y-maze test was performed at day 32 after VCI was induced to evaluate changes in spatial working memory. Experimental animals from both the sham and VCI groups were sacrificed right after the behavioral performance was conducted. A previously reported method was referred to carry out the experiment [49]. The Y-maze apparatus consisted of three arms (1, 2, and 3) that were spaced at 120° angles. Initially, mice were placed in one arm randomly and the sequence and number of arm visits were recorded over an 8-minute period. Spontaneous alternation performance (SAP) and alternating arm return (AAR) % were computed by using the following equation: ((Number of SAP or AAR/(Number of possible alternations) × 100). SAP is an index of spatial working memory. The total number of entries was also recorded. 

### 4.7. Statistical Analysis

All results are expressed as mean ± standard error of mean (S.E.M.). *P* values < 0.05 were considered statistically significant. GraphPad Prism 5.0 (GraphPad, La Jolla, CA, USA) was used for statistical analysis and graphics. A student’s t-test (unpaired, two-tailed) or one-way ANOVA (Tukey correction) was used to investigate the differences between (Sham vs. C57VCI; 5X-Sham vs. 5XVCI) or among the groups (sham vs. C57VCI vs. C57VCI <32 days), respectively.

## Supplementary Materials

Supplementary materials can be found at https://www.mdpi.com/1422-0067/21/8/2820/s1.

## References

[1] Hainsworth A.H., Allan S.M., Boltze J., Cunningham C., Farris C., Head E., Ihara M., Isaacs J.D., Kalaria R.N., Lesnik Oberstein S.A. (2017). Translational models for vascular cognitive impairment: A review including larger species. BMC Med..

[2] Hattori Y., Enmi J., Iguchi S., Saito S., Yamamoto Y., Tsuji M., Nagatsuka K., Kalaria R.N., Iida H., Ihara M. (2016). Gradual Carotid Artery Stenosis in Mice Closely Replicates Hypoperfusive Vascular Dementia in Humans. J. Am. Heart Assoc..

[3] Helman A.M., Murphy M.P. (2016). Vascular cognitive impairment: Modeling a critical neurologic disease in vitro and in vivo. Biochim. Biophys. Acta.

[4] Lee J.H., Kim S.H., Kim G.H., Seo S.W., Park H.K., Oh S.J., Kim J.S., Cheong H.K., Na D.L. (2011). Identification of pure subcortical vascular dementia using 11C-Pittsburgh compound B. Neurology.

[5] Gooch J., Wilcock D.M. (2016). Animal Models of Vascular Cognitive Impairment and Dementia (VCID). Cell Mol. Neurobiol..

[6] Duncombe J., Kitamura A., Hase Y., Ihara M., Kalaria R.N., Horsburgh K. (2017). Chronic cerebral hypoperfusion: A key mechanism leading to vascular cognitive impairment and dementia. Closing the translational gap between rodent models and human vascular cognitive impairment and dementia. Clin. Sci..

[7] Hattori Y., Enmi J., Kitamura A., Yamamoto Y., Saito S., Takahashi Y., Iguchi S., Tsuji M., Yamahara K., Nagatsuka K. (2015). A novel mouse model of subcortical infarcts with dementia. J. Neurosci..

[8] Hattori Y., Kitamura A., Nagatsuka K., Ihara M. (2014). A novel mouse model of ischemic carotid artery disease. PLoS ONE.

[9] Roberts J.M., Maniskas M.E., Bix G.J. (2018). Bilateral carotid artery stenosis causes unexpected early changes in brain extracellular matrix and blood-brain barrier integrity in mice. PLoS ONE.

[10] Nishio K., Ihara M., Yamasaki N., Kalaria R.N., Maki T., Fujita Y., Ito H., Oishi N., Fukuyama H., Miyakawa T. (2010). A mouse model characterizing features of vascular dementia with hippocampal atrophy. Stroke.

[11] Ihara M., Tomimoto H. (2011). Lessons from a mouse model characterizing features of vascular cognitive impairment with white matter changes. J. Aging Res..

[12] Hattori Y., Enmi J., Iguchi S., Saito S., Yamamoto Y., Nagatsuka K., Iida H., Ihara M. (2016). Substantial Reduction of Parenchymal Cerebral Blood Flow in Mice with Bilateral Common Carotid Artery Stenosis. Sci. Rep..

[13] Bordeleau M., ElAli A., Rivest S. (2016). Severe chronic cerebral hypoperfusion induces microglial dysfunction leading to memory loss in APPswe/PS1 mice. Oncotarget.

[14] Park J.H., Hong J.H., Lee S.W., Ji H.D., Jung J.A., Yoon K.W., Lee J.I., Won K.S., Song B.I., Kim H.W. (2019). The effect of chronic cerebral hypoperfusion on the pathology of Alzheimer’s disease: A positron emission tomography study in rats. Sci. Rep..

[15] Lutz C.M., Osborne M.A. (2013). Optimizing mouse models of neurodegenerative disorders: Are therapeutics in sight?. Future Neurol..

[16] Qiu L., Ng G., Tan E.K., Liao P., Kandiah N., Zeng L. (2016). Chronic cerebral hypoperfusion enhances Tau hyperphosphorylation and reduces autophagy in Alzheimer’s disease mice. Sci. Rep..

[17] Kapasi A., Schneider J.A. (2016). Vascular contributions to cognitive impairment, clinical Alzheimer’s disease, and dementia in older persons. Biochim. Biophys. Acta.

[18] Strickland S. (2018). Blood will out: Vascular contributions to Alzheimer’s disease. J. Clin. Investig..

[19] Mullen R.J., Buck C.R., Smith A.M. (1992). NeuN, a neuronal specific nuclear protein in vertebrates. Development.

[20] Yang G., Kitagawa K., Matsushita K., Mabuchi T., Yagita Y., Yanagihara T., Matsumoto M. (1997). C57BL/6 strain is most susceptible to cerebral ischemia following bilateral common carotid occlusion among seven mouse strains: Selective neuronal death in the murine transient forebrain ischemia. Brain Res..

[21] Majid A., He Y.Y., Gidday J.M., Kaplan S.S., Gonzales E.R., Park T.S., Fenstermacher J.D., Wei L., Choi D.W., Hsu C.Y. (2000). Differences in vulnerability to permanent focal cerebral ischemia among 3 common mouse strains. Stroke.

[22] Dichgans M., Leys D. (2017). Vascular Cognitive Impairment. Circ. Res..

[23] Farkas E., Donka G., de Vos R.A., Mihaly A., Bari F., Luiten P.G. (2004). Experimental cerebral hypoperfusion induces white matter injury and microglial activation in the rat brain. Acta Neuropathol..

[24] Fowler J.H., McQueen J., Holland P.R., Manso Y., Marangoni M., Scott F., Chisholm E., Scannevin R.H., Hardingham G.E., Horsburgh K. (2018). Dimethyl fumarate improves white matter function following severe hypoperfusion: Involvement of microglia/macrophages and inflammatory mediators. J. Cereb. Blood Flow Metab..

[25] Ito D., Tanaka K., Suzuki S., Dembo T., Fukuuchi Y. (2001). Enhanced expression of Iba1, ionized calcium-binding adapter molecule 1, after transient focal cerebral ischemia in rat brain. Stroke.

[26] Farkas E., Luiten P.G., Bari F. (2007). Permanent, bilateral common carotid artery occlusion in the rat: A model for chronic cerebral hypoperfusion-related neurodegenerative diseases. Brain Res. Rev..

[27] Shibata M., Ohtani R., Ihara M., Tomimoto H. (2004). White matter lesions and glial activation in a novel mouse model of chronic cerebral hypoperfusion. Stroke.

[28] Jin Z., Jung Y., Yi C.O., Lee J.Y., Jeong E.A., Lee J.E., Park K.J., Kwon O.Y., Lim B.H., Choi N.C. (2018). Atorvastatin pretreatment attenuates kainic acid-induced hippocampal neuronal death via regulation of lipocalin-2-associated neuroinflammation. Korean J. Physiol. Pharmacol..

[29] Lana D., Melani A., Pugliese A.M., Cipriani S., Nosi D., Pedata F., Giovannini M.G. (2014). The neuron-astrocyte-microglia triad in a rat model of chronic cerebral hypoperfusion: Protective effect of dipyridamole. Front. Aging Neurosci..

[30] Broadbent N.J., Squire L.R., Clark R.E. (2004). Spatial memory, recognition memory, and the hippocampus. Proc. Natl. Acad. Sci. USA.

[31] Moser M.B., Moser E.I., Forrest E., Andersen P., Morris R.G. (1995). Spatial learning with a minislab in the dorsal hippocampus. Proc. Natl. Acad. Sci. USA.

[32] Fuchtemeier M., Brinckmann M.P., Foddis M., Kunz A., Po C., Curato C., Dirnagl U., Farr T.D. (2015). Vascular change and opposing effects of the angiotensin type 2 receptor in a mouse model of vascular cognitive impairment. J. Cereb. Blood Flow Metab..

[33] Boehm-Sturm P., Fuchtemeier M., Foddis M., Mueller S., Trueman R.C., Zille M., Rinnenthal J.L., Kypraios T., Shaw L., Dirnagl U. (2017). Neuroimaging Biomarkers Predict Brain Structural Connectivity Change in a Mouse Model of Vascular Cognitive Impairment. Stroke.

[34] Jang H., Kwon H., Yang J.J., Hong J., Kim Y., Kim K.W., Lee J.S., Jang Y.K., Kim S.T., Lee K.H. (2017). Correlations between Gray Matter and White Matter Degeneration in Pure Alzheimer’s Disease, Pure Subcortical Vascular Dementia, and Mixed Dementia. Sci. Rep..

[35] Concha L., Gross D.W., Wheatley B.M., Beaulieu C. (2006). Diffusion tensor imaging of time-dependent axonal and myelin degradation after corpus callosotomy in epilepsy patients. Neuroimage.

[36] Chui H.C., Ramirez-Gomez L. (2015). Clinical and imaging features of mixed Alzheimer and vascular pathologies. Alzheimers Res. Ther..

[37] Attems J., Jellinger K.A. (2014). The overlap between vascular disease and Alzheimer’s disease—Lessons from pathology. BMC Med..

[38] Santos C.Y., Snyder P.J., Wu W.C., Zhang M., Echeverria A., Alber J. (2017). Pathophysiologic relationship between Alzheimer’s disease, cerebrovascular disease, and cardiovascular risk: A review and synthesis. Alzheimers Dement..

[39] Oakley H., Cole S.L., Logan S., Maus E., Shao P., Craft J., Guillozet-Bongaarts A., Ohno M., Disterhoft J., Van Eldik L. (2006). Intraneuronal beta-amyloid aggregates, neurodegeneration, and neuron loss in transgenic mice with five familial Alzheimer’s disease mutations: Potential factors in amyloid plaque formation. J. Neurosci..

[40] Yamada M., Ihara M., Okamoto Y., Maki T., Washida K., Kitamura A., Hase Y., Ito H., Takao K., Miyakawa T. (2011). The influence of chronic cerebral hypoperfusion on cognitive function and amyloid beta metabolism in APP overexpressing mice. PLoS ONE.

[41] Salvadores N., Searcy J.L., Holland P.R., Horsburgh K. (2017). Chronic cerebral hypoperfusion alters amyloid-beta peptide pools leading to cerebral amyloid angiopathy, microinfarcts and haemorrhages in Tg-SwDI mice. Clin. Sci..

[42] Walha K., Ricolfi F., Bejot Y., Nonent M., Ben Salem D. (2013). Hippocampus: A “forgotten” border zone of brain ischemia. J. Neuroimaging.

[43] Gubskiy I.L., Namestnikova D.D., Cherkashova E.A., Chekhonin V.P., Baklaushev V.P., Gubsky L.V., Yarygin K.N. (2018). MRI Guiding of the Middle Cerebral Artery Occlusion in Rats Aimed to Improve Stroke Modeling. Transl. Stroke Res..

[44] Erdem A., Yasargil G., Roth P. (1993). Microsurgical anatomy of the hippocampal arteries. J. Neurosurg..

[45] Akiyama H., McGeer P.L. (2004). Specificity of mechanisms for plaque removal after A beta immunotherapy for Alzheimer disease. Nat. Med..

[46] Pontarelli F., Ofengeim D., Zukin R.S., Jonas E.A. (2012). Mouse Transient Global Ischemia Two-Vessel Occlusion Model. Bio Protoc..

[47] Lee N.K., Kim H.S., Yoo D., Hwang J.W., Choi S.J., Oh W., Chang J.W., Na D.L. (2017). Magnetic Resonance Imaging of Ferumoxytol-Labeled Human Mesenchymal Stem Cells in the Mouse Brain. Stem Cell Rev. Rep..

[48] Lee N.K., Yang J., Chang E.H., Park S.E., Lee J., Choi S.J., Oh W., Chang J.W., Na D.L. (2016). Intra-Arterially Delivered Mesenchymal Stem Cells Are Not Detected in the Brain Parenchyma in an Alzheimer’s Disease Mouse Model. PLoS ONE.

[49] Lee G.Y., Lee C., Park G.H., Jang J.H. (2017). Amelioration of Scopolamine-Induced Learning and Memory Impairment by alpha-Pinene in C57BL/6 Mice. Evid. Based Complement. Alternat. Med..

