# Supplementary material for: Heterogeneous Disease Progression in a Mouse Model of Vascular Cognitive Impairment
Source: Int J Mol Sci. 2020 Apr 17;21(8):2820. doi: 10.3390/ijms21082820 (PMC7215687; doi:10.3390/ijms21082820)
Supplement: Supplementary file 1 [file ijms-21-02820-s001.pdf]

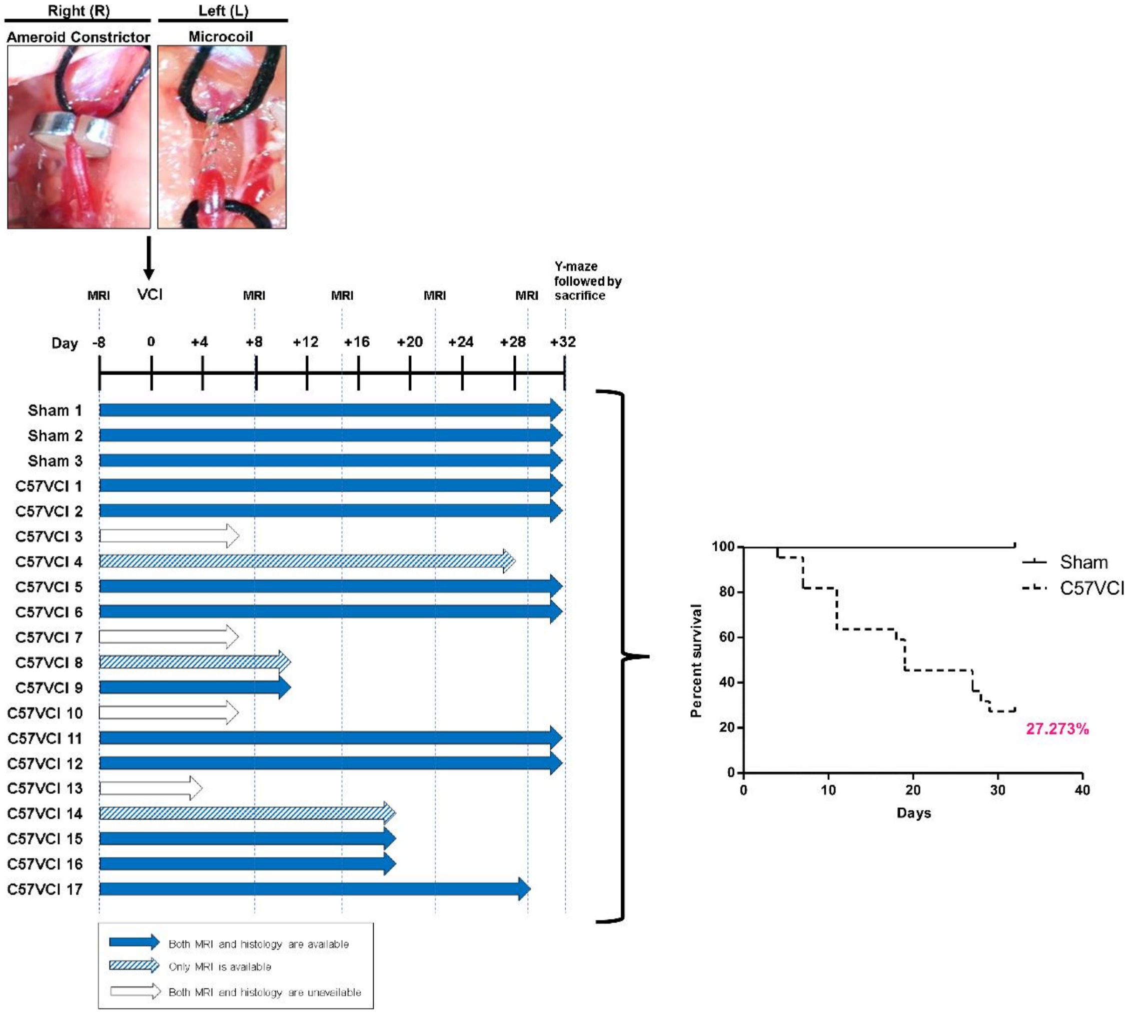

Figure S1. C57VCI experimental design. Timeline of the experiment for C57VCI mice. VCI was induced by placing a microcoil on the left (L) CCA and an ameroid constrictor on the right (R) CCA. MR images were acquired weekly up to 4 weeks (Day 8, 15, 22, and 29) following VCI surgery. Changes in spatial working memory (Y-maze) was assessed before sacrificing the mice at Day 32. Solid arrows represent mice where MR images and histological data are both available. Arrows with diagonal stripes indicate mice where only MR images are available because they died before Day 32. These mice died overnight and thus the brain tissues were too degraded and fragile to undergo histological processing. For mice labeled with empty arrows, both MR images and histological data are unavailable because the mice died before 1 week (first follow-up MRI). The Kaplan-Meier survival curve (lower right) illustrates the high mortality rate of the C57VCI group.

Table S1. Pathologic features and analysis of C57BL6/J mice that underwent VCI surgery

Experimental Animal	Survived up to (# of days)	Y-maze	DTI analysis	Infarct		Hippocampal Neuronal Loss		Hippocampal area (IHC)		
				Left	Right	Left	Right	NeuN	Iba-1	GFAP
Sham 1	32	●	●	-	-	-	-	●	●	●
Sham 2	32	●	●	-	-	-	-	●	●	●
Sham 3	32	●	●	-	-	-	-	●	●	●
C57VCI 1	32	●	●	-	-	-	-	●	●	●
C57VCI 2	32	●	●	-	-	-	●	●	●	●
C57VCI 3	7	-	-	N/A		N/A		-	-	-
C57VCI 4	28	-	N/A	-	●	N/A		-	-	-
C57VCI 5	32	●	●	●	-	●	-	●	●	●
C57VCI 6	32	●	●	-	-	-	●	●	●	●
C57VCI 7	7	-	-	N/A		N/A		-	-	-
C57VCI 8	11	-	-	N/A		N/A		-	-	-
C57VCI 9	11	-	N/A	-	●	N/A		-	-	-
C57VCI 10	7	-	-	N/A		N/A		-	-	-
C57VCI 11	32	●	●	-	-	-	●	●	●	●
C57VCI 12	32	●	●	-	-	-	-	●	●	●
C57VCI 13	4	-	-	N/A		N/A		-	-	-
C57VCI 14	19	-	-	N/A		N/A		-	-	-
C57VCI 15	19	-	●	●	-	●	-	-	-	-
C57VCI 16	19	-	●	-	●	-	-	-	-	-
C57VCI 17	29	-	●	●	●	●	-	-	-	-

Abbreviation: N/A = Not applicable.

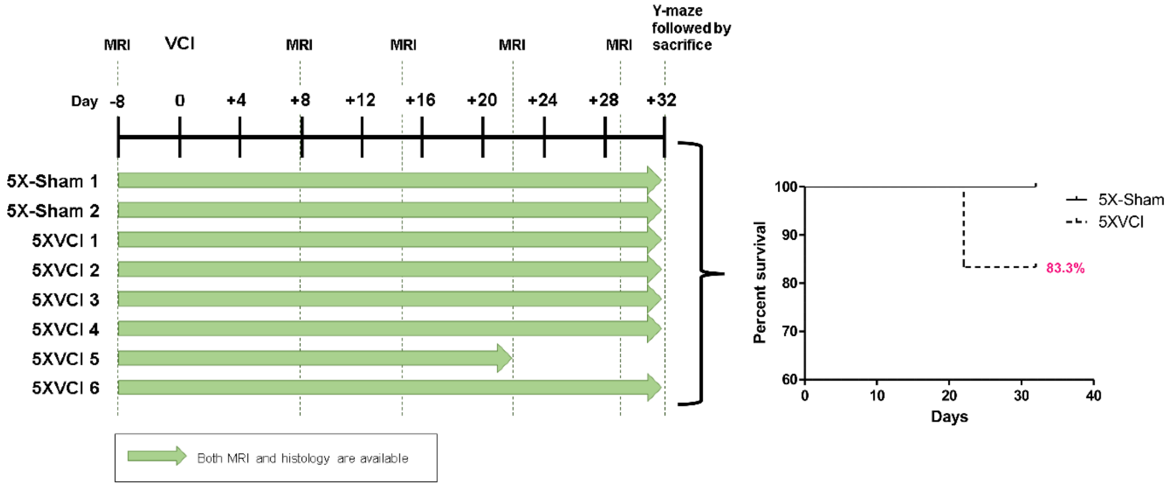

Figure S2. 5XVCI experimental design. Timeline of the experiment for 5XVCI mice. MR images were acquired weekly up to 4 weeks following VCI surgery. Changes in spatial working memory (Y-maze) was assessed before sacrificing the mice at Day 32. MR images and histological data were available for all the 5X-Sham (n=2) and 5XVCI (n=6) mice (solid arrows). The survival rate (Kaplan-Meier survival curve) of the 5XVCI mice is shown on the lower right.

Table S2. Pathologic features and analysis of 5XFAD mice that underwent VCI surgery

Experimental Animal	Survived up to (# of days)	Y-maze	Infarct		Hippocampal Neuronal Loss		Hippocampal area (IHC)			
			Left	Right	Left	Right	NeuN	Iba-1	GFAP	Aβ
5X-Sham 1	32	●	-	-	-	-	●	●	●	●
5X-Sham 2	32	●	-	-	-	-	●	●	●	●
5XVCI 1	32	●	-	-	-	●	●	●	●	●
5XVCI 2	32	●	-	-	-	-	●	●	●	●
5XVCI 3	32	●	-	-	-	-	●	●	●	●
5XVCI 4	32	●	-	-	-	●	●	●	●	●
5XVCI 5	22	-	●	-	-	●	-	-	-	-
5XVCI 6	32	●	-	●	-	-	●	●	●	●
